# Host species identity outweighs life form in shaping the root endophytic bacterial communities of 10 *Cymbidium* species grown under uniform conditions

**DOI:** 10.3389/fmicb.2026.1996961

**Published:** 2026-10-29

**Authors:** Xueda Chen, Rui Fang, Tinghua Zhang, Guanfei Zhao, Fang Wang

**Affiliations:** 1 College of Forestry and Grassland, The Orchid Conservation Center of Xizang, Xizang Agriculture and Animal Husbandry University, Linzhi, China; 2 Vegetable Research Institute, Xizang Agriculture and Animal Husbandry Science Academy, Lhasa, China

**Keywords:** community assembly, *Cymbidium*, host-species identity, life form, root endophytic bacteria

## Abstract

Research on orchid microbiomes has long been dominated by mycorrhizal fungi, whereas the community structure of orchid-associated bacteria remains poorly understood, particularly across host life forms. We compared the root endophytic bacterial communities of 10 *Cymbidium* species (six epiphytic and four terrestrial), all cultivated in the same greenhouse. Communities were profiled by 16S rRNA gene amplicon sequencing, with six biological replicates per species. After rarefaction to 29,628 sequences per sample, we obtained 129,922 amplicon sequence variants (ASVs) assigned to 33 phyla and 779 genera. Proteobacteria dominated the communities (mean relative abundance 83.4%), followed by Actinobacteriota and Acidobacteriota; the most abundant genera were *Pseudomonas, Sphingomonas*, the *Burkholderia–Caballeronia–Paraburkholderia* complex, *Bradyrhizobium* and *Reyranella*. Only seven ASVs were shared by all 10 species (each detected in at least one replicate sample of every species), and only one ASV occurred in all 60 samples, indicating an extremely small interspecific core microbiome. No alpha-diversity index differed between life forms. Among host species, however, the Shannon, Simpson, and Pielou evenness indices differed significantly among species. Single-factor (marginal) permutational multivariate analysis indicated a hierarchy of host effects: life form explained only 6.9% of community variation, infrageneric section 35.6%, and host species 59.9%, with significant differentiation even among species within the same section (*R*^2^ ≈ 0.51); because life form, infrageneric section and host species are partially confounded in this design, these values describe associations rather than fully independent effects. Random forest classification independently corroborated this hierarchy. Community composition correctly assigned all 60 samples to their host species, whereas life-form prediction did not generalize across species. Under leave-one-species-out cross-validation, life-form accuracy fell to 50.0%, below the 60% majority-class baseline. LEfSe analysis identified 279 biomarker taxa spanning all 10 species. Predicted functional profiles were dominated by biosynthesis pathways (69.8%) and were largely stable between life forms. The neutral community model explained little of the variation in ASV occurrence frequency (*R*^2^ = 0.035). This indicates that neutral dispersal alone cannot account for community assembly. The normalized stochasticity ratio averaged 0.576, only slightly above the 0.5 threshold for stochastic dominance, pointing to predominantly deterministic assembly with a secondary stochastic contribution. Within the limits of this partially confounded design, these results indicate that, with environmental heterogeneity minimized, host species identity is a stronger correlate of root endophytic bacterial community differentiation than life form in *Cymbidium*, providing a basis for the species-specific utilization of beneficial endophytes in orchid cultivation and conservation.

## 1 Introduction

*Cymbidium* Sw. is among the most ornamental and economically important genera of Orchidaceae. China is a major center of its distribution and diversity, harboring 57 species (Yang et al., 2023; Zhou et al., 2021). The traditional Chinese orchids (e.g., *C. goeringii, C. faberi, C. ensifolium, C. sinense*, and *C. kanran*) all belong to this genus, which has a long history of cultivation and supports a large-scale floriculture industry (Yang et al., 2023). The life forms of *Cymbidium* species are diverse, including terrestrial, epiphytic and mycoheterotrophic types, and species within the same genus differ markedly in habitat adaptation and root morphology (Kim and Chase, 2017; Motomura et al., 2010). The genus therefore offers a valuable system for studying how host life form and phylogenetic position relate to the associated microbial communities. Notably, when cultivated under identical environmental conditions, this genus enables us to disentangle intrinsic host-related effects from confounding environmental variation.

Plant endophytic bacteria colonize the internal tissues of healthy plants without causing visible disease symptoms and constitute an important component of the plant microbiome (Hardoim et al., 2015). They can promote host growth and stress tolerance through auxin production, nitrogen fixation, phosphate solubilization, ACC deaminase synthesis and pathogen antagonism, while also participating in nutrient cycling and the regulation of secondary metabolism (Hardoim et al., 2015; Afzal et al., 2019). As the primary organ of material exchange between the plant and its environment, the root harbors an endophytic bacterial community jointly shaped by host selection and environmental microbial source pools. Disentangling the drivers governing such community assembly remains a central question in plant-microbe interaction research (Afzal et al., 2019).

Orchid seeds are minute and lack endosperm; under natural conditions, their germination and seedling establishment depend largely on mycorrhizal fungi, and orchid mycorrhizae have accordingly remained the main focus of research in this field (Li et al., 2021). Studies of orchid-associated bacteria started later, yet the available evidence indicates that bacteria also play important roles: orchid roots can be colonized by diverse bacterial taxa, some of which produce indole-3-acetic acid (IAA) and promote seed germination and seedling growth (Kaur and Sharma, 2021; Tsavkelova et al., 2006, 2007), and endophytic bacteria with auxin-synthesizing and growth-promoting potential have also been confirmed in the aerial roots of epiphytic orchids (Yadav et al., 2022). These findings suggest that the diversity and function of orchid root-associated bacteria may have been grossly underestimated (Kaur and Sharma, 2021). However, most existing studies are based on the screening of culturable strains from a single species. Community-level studies based on high-throughput sequencing remain scarce, and the few available ones have mostly sampled wild-grown plants, in which heterogeneous field environments confound host effects. A systematic understanding of how bacterial communities vary with host species and life form (epiphytic vs. terrestrial) under controlled, common-garden conditions is therefore still lacking.

*Cymbidium* is one of the better-studied orchid genera, yet its microbiome research is likewise dominated by mycorrhizal fungi (Ogura-Tsujita et al., 2012). On the bacterial side, only scattered reports are available: a survey of the rhizosphere soil microbial diversity of introduced *C. goeringii* and *C. faberi* (Lv et al., 2023), the screening of growth-promoting bacteria for *Cymbidium* (Gontijo et al., 2018), and the isolation of phosphate-solubilizing endophytic bacteria from the roots of Chinese *Cymbidium* orchids (Sun et al., 2025). To our knowledge, no comparative study has yet examined the root endophytic bacterial communities of multiple *Cymbidium* species spanning life forms. This gap limits our understanding of *Cymbidium*-bacteria interactions and hinders the utilization of endophytic bacterial resources for orchid cultivation and conservation.

In this study, we compared the root endophytic bacterial communities of 10 *Cymbidium* species (six epiphytic and four terrestrial; six replicates each) grown in the same greenhouse, using 16S rRNA gene high-throughput sequencing, in order to address four questions: (1) how do community composition and diversity differ among life forms, infrageneric sections and host species; (2) which bacterial taxa serve as biomarkers of each species; (3) what is the predicted functional potential of these communities; and (4) what are the relative roles of deterministic selection and stochastic processes in community assembly, as assessed by niche breadth analysis, the neutral community model (NCM) and the normalized stochasticity ratio (NST)? The results provide a theoretical basis for mining beneficial endophytic bacteria, developing microbial inoculants, and supporting *ex situ* conservation and artificial propagation of *Cymbidium*.

## 2 Materials and methods

### 2.1 Plant materials, sample collection, and surface sterilization

Ten *Cymbidium* species cultivated in the greenhouse of Xizang Agriculture and Animal Husbandry University (Linzhi, Xizang, China) were used, comprising six epiphytic and four terrestrial species. All plants had been transplanted into the same greenhouse and grown in the same substrate under identical management for more than 5 years. The cultivation substrate consisted of bark, coconut coir, perlite and compost mixed at a ratio of 2:2:1:5, with routine water and fertilizer management. The 10 *Cymbidium* species were assigned to infrageneric sections according to the plastid phylogenomic classification of Chen et al. (2024). Details of the plant materials are listed in Table 1. All plants were originally collected from natural populations in Xizang and had been maintained in the greenhouse for more than 5 years before sampling; they were not derived from tissue culture. This choice was deliberate: our aim was to characterize the root endophytic communities of cultivated *Cymbidium* as they are actually managed in conservation and horticultural practice, and the prolonged common-garden cultivation allowed the communities to re-assemble under a shared environment, so that the remaining differentiation can be attributed primarily to the host.

**Table 1:** Information of the *Cymbidium* materials used in this study.

Code	Species	Life form	Section	Replicates
CD	*Cymbidium devonianum*	Epiphytic	Bigibbarium	6
CT	*Cymbidium tigrinum*	Epiphytic	Parishiella	6
CE	*Cymbidium elegans*	Epiphytic	Cyperochis	6
CI	*Cymbidium iridioides*	Epiphytic	Cyperochis	6
CM	*Cymbidium mastersii*	Epiphytic	Cyperochis	6
CY	*Cymbidium mannii*	Epiphytic	Cymbidium	6
CC	*Cymbidium cyperifolium*	Terrestrial	JensoaII	6
CF	*Cymbidium faberi*	Terrestrial	JensoaI	6
CG	*Cymbidium goeringii*	Terrestrial	JensoaI	6
CL	*Cymbidium lancifolium*	Terrestrial	Pachyrhizanthe	6

Section assignment follows Chen et al. (2024).

Root samples were collected in June 2025. For each species, six healthy, pest-free plants were selected as biological replicates, with each replicate corresponding to one independent plant grown in its own pot (six plants in six separate pots per species, one root sample per plant). Plants were removed from their pots, loosely attached substrate was shaken off, and healthy roots were excised into sterile bags, transported to the laboratory on ice, and surface-sterilized within 4 h.

Roots were first rinsed under tap water to remove debris and washed three times with sterile water, then cut into 1–2 cm segments. Surface sterilization comprised successive immersions in 75% ethanol for 30 s, sodium hypochlorite solution (1% available chlorine) for 2 min, and 75% ethanol again for 30 s, followed by five rinses in sterile water. As a sterility check, 100 μL of the final rinse was spread onto LB agar and incubated at 28 °C for 3 days; the absence of colonies was taken as evidence of complete surface sterilization. Samples showing bacterial colony growth on LB plates were discarded and not subjected to DNA extraction. Sterilized roots were blotted dry with sterile filter paper, flash-frozen in liquid nitrogen, and stored at −80 °C until use.

### 2.2 DNA extraction, PCR amplification, and high-throughput sequencing

Total DNA was extracted from the root samples using the CTAB method (Doyle and Doyle, 1987). DNA quantity and quality were assessed by 0.8% agarose gel electrophoresis and with a NanoDrop NC2000 spectrophotometer (Thermo Fisher Scientific, Waltham, MA, USA). The V5–V7 region of the bacterial 16S rRNA gene was amplified with barcoded universal primers (F: 5′-AACMGGATTAGATACCCKG-3′; R: 5′-ACGTCATCCCCACCTTCC-3′). Each 20 μL PCR mixture contained 0.25 μL of Q5 high-fidelity DNA polymerase, 5 μL of 5 × reaction buffer, 5 μL of 5 × High GC buffer, 2 μL of dNTPs (10 mM), 1 μL (10 μM) of each forward and reverse primer, 2 μL of DNA template, and ddH2O to volume. Cycling conditions were 98 °C for 5 min; 25 cycles of 98 °C for 30 s, 52 °C for 30 s and 72 °C for 45 s; and a final extension at 72 °C for 5 min. Amplicons were checked on 2% agarose gels, gel-purified, pooled in equimolar amounts, and sequenced on an Illumina NovaSeq 6000 platform (500 cycles) in paired-end 250 bp mode.

### 2.3 Sequence processing and taxonomic annotation

Bioinformatic analyses were performed on the QIIME 2 platform (v2024.5) (Bolyen et al., 2019), following the official tutorials with minor modifications. Raw data were demultiplexed with the demux plugin, primer sequences were trimmed with the cutadapt plugin (Martin, 2011), and quality filtering, denoising, paired-end merging and chimera removal were performed with the DADA2 plugin (Callahan et al., 2016), yielding amplicon sequence variants (ASVs). Taxonomy was assigned against the SILVA 138 database (Quast et al., 2013) using the pretrained Naive Bayes classifier in q2-feature-classifier (classify-sklearn, default parameters; Bokulich et al., 2018). Sequencing depth varied among samples. To keep all 60 samples in the analysis, we rarefied each one to the smallest per-sample read count. Bar plots of community composition at each taxonomic level were then drawn in R v4.3.3.

### 2.4 Statistical analysis

Alpha-diversity indices (Chao1, observed ASVs, Shannon, Simpson, Pielou's evenness, and Goods coverage) were compared across life forms, infrageneric groups and species by one-way ANOVA. Where ANOVA indicated significance, group means were separated with Tukey's honestly significant difference (HSD) *post-hoc* test. Beta diversity was computed from Bray-Curtis distance matrices and visualized by principal coordinates analysis (PCoA); the effects of life form, infrageneric group and host species on community structure were tested with permutational multivariate analysis of variance (PERMANOVA, adonis, 999 permutations; Anderson, 2001) based on Bray-Curtis dissimilarity matrices. Each factor was tested in a separate single-factor model. The *R*^2^ values in Table 3 are therefore marginal effects, each quantifying the share of community variation associated with that factor alone. They are not additive. We did not fit a joint sequential model, because the three factors are not orthogonal in this design: every species represents a single life form, and several sections contain species of only one life form (Table 1). Their independent effects cannot therefore be partitioned unambiguously. This partial confounding is accounted for when interpreting the results (see Sections 3.4 and 4.2). Biomarker bacteria enriched in each species were identified by linear discriminant analysis effect size (LEfSe; Segata et al., 2011), with species as the grouping factor (LDA > 2, *P* < 0.05).

Random forest classification (Breiman, 2001) was used to evaluate how well root endophytic bacterial community composition predicts host species identity and life form. All models were fitted in R (version 4.3.3) using the classif.ranger learner of the mlr3 framework (version 0.22.0; Lang et al., 2019), which implements random forests through the ranger package (Wright and Ziegler, 2017), with 500 classification trees and the number of predictor variables sampled at each split set to the square root of the total number of features. Models were built on the rarefied ASV feature table (129,922 ASVs × 60 samples, 29,628 sequences per sample), the same table used for the diversity analyses. For host-species classification, species was set as the grouping factor, predictive performance was evaluated from the out-of-bag (OOB) error, and ASVs were ranked by feature importance. For life-form classification, life form was set as the grouping factor, and performance was assessed from the OOB error together with nested stratified cross-validation, in which hyperparameters were selected within an inner resampling loop and predictive performance was estimated in an outer loop. Because each species represents a single life form, retaining replicates of the same species in both training and test sets can inflate the apparent life-form classification accuracy; leave-one-species-out cross-validation (LOGO-CV) was therefore additionally performed, in which all six replicates of a given species were withheld and their life form was predicted from models trained on the remaining nine species. The complete random forest results are provided in Supplementary Table S2.

Functional potential was predicted with PICRUSt2 (Douglas et al., 2020) from the 16S rRNA sequences and summarized at pathway level against the MetaCyc database (Caspi et al., 2020), with KEGG ortholog results provided as supplementary tables. Community assembly was examined in three ways: Levins' niche breadth per ASV (simple and abundance-weighted); the Sloan neutral community model (NCM; Sloan et al., 2006) fitted to the relationship between ASV occurrence frequency and relative abundance, in which *R*^2^ estimates the share explained by neutral (stochastic) processes and Nm the community-level migration rate; and the normalized stochasticity ratio (NST; Ning et al., 2019) for the relative weight of deterministic vs. stochastic processes. Functional groups were predicted with FAPROTAX (Louca et al., 2016) and expressed as a percentage of the assigned pool in each sample. Differences were tested with Mann–Whitney *U*-tests (life form) and Kruskal–Wallis tests (species), both Benjamini–Hochberg corrected across all annotated groups. All analyses were performed in R v4.3.3 (vegan, picante and ggplot2 packages); multiple testing was corrected with the Benjamini–Hochberg method, and significance was set at *P* < 0.05.

## 3 Results

### 3.1 Sequencing overview and ASV annotation

The 60 root samples (10 species × 6 replicates) yielded 6,420,795 raw reads. Quality filtering, paired-end merging, chimera removal and the elimination of singletons left 2,576,775 high-quality sequences—an average of 42,946 per sample, ranging from 31,188 to 55,052. After rarefaction to 29,628 sequences per sample, the data resolved into 129,922 ASVs assigned to 33 phyla, 91 classes, 218 orders, 377 families, 779 genera, and 1,420 species. Goods coverage ranged from 97.43% to 99.76%, and rarefaction curves flattened with increasing sequencing depth (Figure 1), indicating that the great majority of bacterial taxa had been captured. ASV counts per species ranged from 10,201 (*Cymbidium mannii*) to 16,081 (*Cymbidium devonianum*). The overlap across species was very small: only seven ASVs were shared by all 10 species, and a single ASV was present in all 60 samples. Here, an ASV was considered shared by all 10 species if it was detected in at least one replicate sample of every species; occurrence in all 60 samples was not required. The seven ASVs shared by all 10 host species were all affiliated with Proteobacteria, comprising *Pseudomonas* (ASV_86589), *Bradyrhizobium* (ASV_22104, ASV_35699), *Reyranella* (ASV_16347), *Burkholderia-Caballeronia-Paraburkholderia* (ASV_73514), an unclassified member of Comamonadaceae (ASV_154881), and *Roseiarcus* (ASV_115365) (Supplementary Table S1). The single ASV detected in all 60 samples was ASV_22104 (*Bradyrhizobium*), with a mean relative abundance of 0.71%. Thus, although ubiquitous, this shared ASV remained at low abundance, suggesting that the common core taxa are widespread but not dominant within the communities.

**Figure 1:**
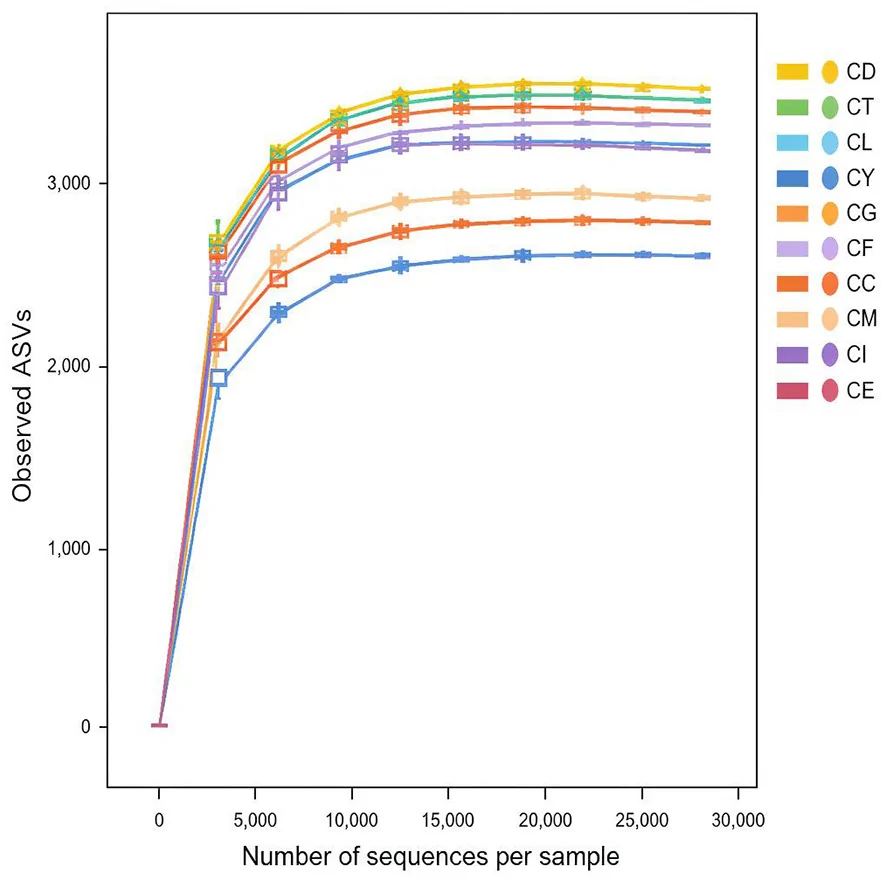
Rarefaction curves of observed ASVs for the 60 root samples, colored by host species. All curves approach a plateau with increasing sequencing depth, indicating that the great majority of bacterial taxa had been captured.

### 3.2 Community composition of root endophytic bacteria

Proteobacteria dominated at the phylum level (mean relative abundance 83.4%), followed by Actinobacteriota (10.3%), Acidobacteriota (2.5%), unclassified bacteria (1.1%), and Firmicutes (0.8%; Figure 2). Epiphytic species harbored a lower relative abundance of Proteobacteria than terrestrial ones (80.7% vs. 87.4%) but higher Actinobacteriota (12.7% vs. 6.8%) and Acidobacteriota (2.9% vs. 1.8%); none of these contrasts was significant at the species-mean level (*P* > 0.05), and they are therefore reported here as trends. Dominant phyla also differed among infrageneric sections: Actinobacteriota, for instance, reached 15.7% in the Cyperochis group vs. 6.3% in the Jensoa group. Species-level differences were wider: Proteobacteria ranged from 58.4% (*Cymbidium elegans*) to 95.6% (*Cymbidium mannii*), and Actinobacteriota from 2.2% (*Cymbidium mannii*) to 28.0% (*Cymbidium elegans*), highlighting strong host-species filtering on root-endophytic bacterial phyla. At the genus level, the most abundant taxa were *Pseudomonas* (7.6%), *Sphingomonas* (6.2%), the *Burkholderia–Caballeronia–Paraburkholderia* complex (6.1%), *Bradyrhizobium* (5.0%) and *Reyranella* (4.6%).

**Figure 2:**
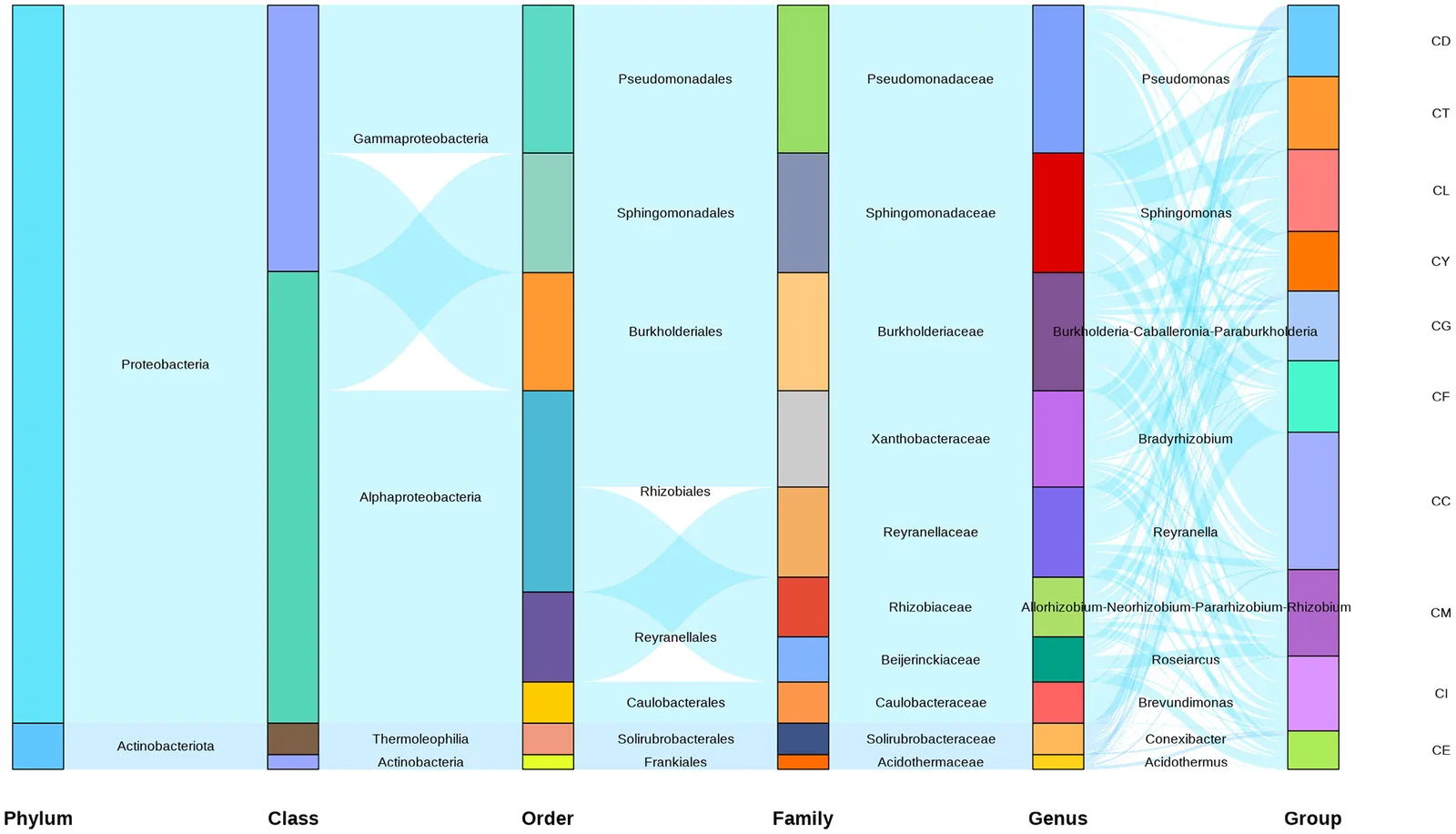
Community composition of root endophytic bacteria at the phylum and genus levels across the 10 *Cymbidium* species (samples grouped by host species).

### 3.3 Alpha diversity among life forms, infrageneric groups, and species

No alpha-diversity index differed between epiphytic and terrestrial species (*P* > 0.05). Chao1 (3,165.75 vs. 3,164.01) and Shannon (9.51 vs. 9.59) values were almost identical between the two life forms (Table 2, Figure 3). Among the six infrageneric groups, Chao1 did not differ significantly among groups (*F* = 1.99, *P* = 0.095), whereas Shannon (*F* = 3.98, *P* = 0.004), Simpson (*F* = 3.66, *P* = 0.006), and Pielou's evenness (*F* = 3.89, *P* = 0.004) did. Among species, *Cymbidium devonianum* harbored the highest richness and diversity (Chao1 3,506.44 ± 169.25; Shannon 10.18 ± 0.07), *Cymbidium mannii* the lowest richness (Chao1 2,603.96 ± 198.49), and *Cymbidium cyperifolium* the lowest diversity (Shannon 8.38 ± 0.45). One-way ANOVA showed that Chao1 did not differ significantly among species (*F* = 1.87, *P* = 0.079), whereas Shannon (*F* = 8.32, *P* < 0.001), Simpson (*F* = 5.14, *P* < 0.001), and Pielou's evenness (*F* = 9.53, *P* < 0.001) differed highly significantly; observed ASVs also differed significantly (*F* = 2.61, *P* = 0.015). Because richness (Chao1) did not differ significantly among species and each species was represented by six replicates, the ranking of species by mean richness and diversity given above is presented as descriptive only and does not imply significant contrasts between particular species.

**Table 2:** Alpha diversity indices of root endophytic bacteria among life forms and *Cymbidium* species (mean ± SE).

Life form/Species	Chao1	Shannon	Observed ASVs	Goods coverage/%
Epiphytic	3,165.75 ± 96.46	9.51 ± 0.14	3,028.39 ± 89.00	98.63 ± 0.10
*Cymbidium devonianum*	3,506.44 ± 169.25	10.18 ± 0.07	3,358.33 ± 136.64	98.49 ± 0.23
*Cymbidium tigrinum*	3,440.54 ± 212.80	10.00 ± 0.09	3,305.83 ± 173.86	98.60 ± 0.29
*Cymbidium elegans*	3,371.82 ± 160.27	9.96 ± 0.17	3,259.00 ± 141.92	98.70 ± 0.16
*Cymbidium iridioides*	3,162.26 ± 253.15	9.56 ± 0.21	3,037.50 ± 215.37	98.73 ± 0.36
*Cymbidium mastersii*	2,909.48 ± 254.20	8.88 ± 0.28	2,753.83 ± 228.72	98.54 ± 0.27
*Cymbidium mannii*	2,603.96 ± 198.49	8.48 ± 0.39	2,455.83 ± 191.05	98.72 ± 0.14
Terrestrial	3,164.01 ± 119.59	9.59 ± 0.19	3,040.33 ± 105.37	98.77 ± 0.15
*Cymbidium cyperifolium*	2,785.10 ± 321.41	8.38 ± 0.45	2,645.67 ± 287.26	98.77 ± 0.33
*Cymbidium faberi*	3,304.66 ± 193.71	10.08 ± 0.18	3,175.67 ± 160.57	98.73 ± 0.28
*Cymbidium goeringii*	3,369.02 ± 246.93	9.96 ± 0.13	3,232.67 ± 196.86	98.66 ± 0.38
*Cymbidium lancifolium*	3,197.27 ± 141.61	9.96 ± 0.14	3,107.33 ± 118.92	98.93 ± 0.22

**Figure 3:**
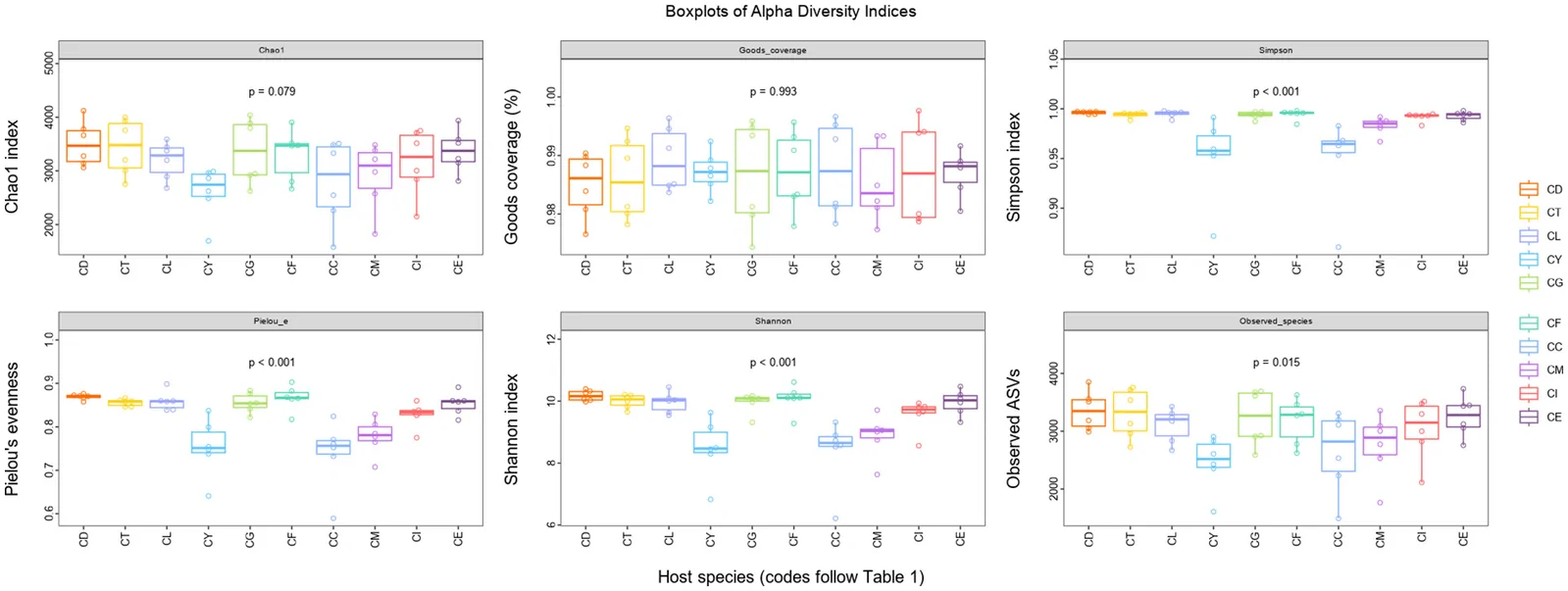
Comparison of alpha-diversity indices (Chao1, observed ASVs, Shannon, Simpson, Pielou's evenness and Goods coverage) among the 10 *Cymbidium* species (box plots; significance by one-way ANOVA with Tukey's HSD *post-hoc* test).

### 3.4 Differentiation of community structure (beta diversity)

Principal coordinates analysis based on Bray–Curtis distances showed that the 60 samples clustered primarily by host species, with only a slight tendency toward separation between epiphytic and terrestrial life forms; the first and second ordination axes explained 10.5% and 8.7% of the total variation, respectively (Figure 4). Single-factor PERMANOVA models, which estimate the marginal effect of each factor (Table 3) indicated that life form had a significant but weak effect on community structure (*R*^2^ = 0.069, *F* = 4.27, and *P* = 0.001), that infrageneric group had a highly significant effect (*R*^2^ = 0.356, *F* = 5.98, and *P* = 0.001), and that host species had the strongest effect, explaining 59.9% of the total community variation (*R*^2^ = 0.599, *F* = 8.28, and *P* = 0.001). Because life form, infrageneric group and host species are partially confounded in this design, these marginal *R*^2^ values are not additive and should not be read as independent contributions; in particular, the species-level estimate subsumes variation that is also structured by life form and section. Community structure still differed significantly among species within the same infrageneric group (Jensoa group: *R*^2^ = 0.506, *F* = 7.68, and *P* = 0.001; Cyperochis group: *R*^2^ = 0.509, *F* = 7.78, and *P* = 0.001). At the genus level, 321 of the 590 tested taxa differed significantly among species (adjusted *P* < 0.05); the most strongly differentiated taxa included *Skermanella, Herbaspirillum, Alsobacter, Lautropia* and *Ochrobactrum* (adjusted *P* < 1.0 × 10^−6^). Community typing (Arumugam et al., 2011) resolved the 60 samples into two clusters (optimal *K* = 2 by the CH index; 54 and 6 samples, respectively). The minority cluster comprised only samples of terrestrial species: five of the six replicates of *Cymbidium cyperifolium* and one replicate of *Cymbidium faberi* were assigned to Cluster 2, whereas all six replicates of the remaining eight species belonged to the majority cluster (Supplementary Figure S3).

**Figure 4:**
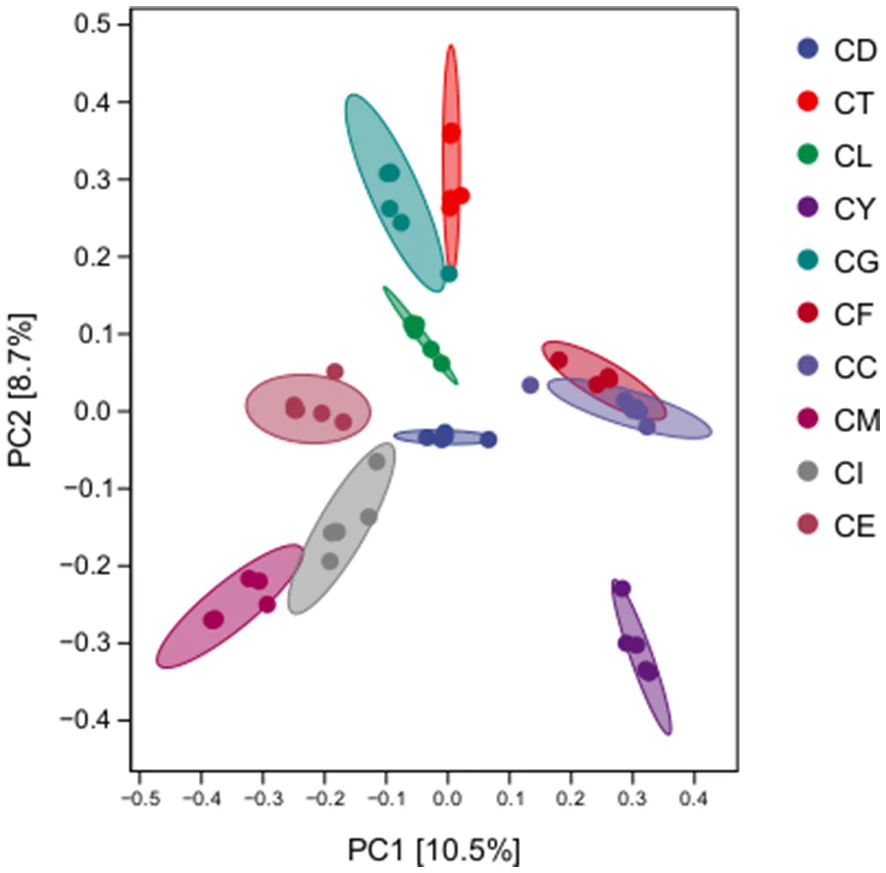
Principal coordinates analysis (PCoA) of root endophytic bacterial communities based on Bray–Curtis distances at the ASV level (PC1 = 10.5%, PC2 = 8.7%). Points are colored by host species; ellipses indicate 95% confidence intervals for each species.

**Table 3:** PERMANOVA of the effects of life form, infrageneric group (section) and host species on root endophytic bacterial community structure.

Factor	df	F	*R* ^2^	*P*
Life form	1	4.27	0.069	0.001
Infrageneric group (section)	5	5.98	0.356	0.001
Host species	9	8.28	0.599	0.001

Values are marginal effects, each obtained from a separate single-factor PERMANOVA model (999 permutations, Bray-Curtis distances). Because life form, infrageneric group and host species are partially confounded in this design, the R^2^ values are not additive and do not represent independent contributions.

### 3.5 Biomarker bacteria of each species

LEfSe analysis (LDA > 2, *P* < 0.05, grouped by sample) returned 279 biomarker taxa in total: 192 for the six epiphytic species and 87 for the four terrestrial species. The numbers per species were 58 (*Cymbidium elegans*), 43 (*C. devonianum*), 34 (*C. faberi*), 30 (*C. iridioides*), 26 (*C. tigrinum*), 24 (*C. cyperifolium*), 22 (*C. goeringii*), 20 (*C. mannii*), 15 (*C. mastersii*), and 7 (*C. lancifolium*; Figure 5). The taxa with the highest LDA scores in each species were: *Pseudomonas* in *C. cyperifolium* (LDA = 5.40); Proteobacteria and Xanthomonadales in *C. mannii* (5.26 and 5.23); Sphingomonadales in *C. tigrinum* (5.10); Rhizobiales in *C. devonianum* (5.07); Burkholderiaceae in *C. mastersii* (5.05); Caulobacterales in *C. faberi* (4.97); Acidimicrobiia in *C. elegans* (4.85); an unclassified Alphaproteobacteria lineage in *C. goeringii* (4.64); an unassigned Gammaproteobacteria order in *C. iridioides* (4.62); and Comamonadaceae in *C. lancifolium* (4.28).

**Figure 5:**
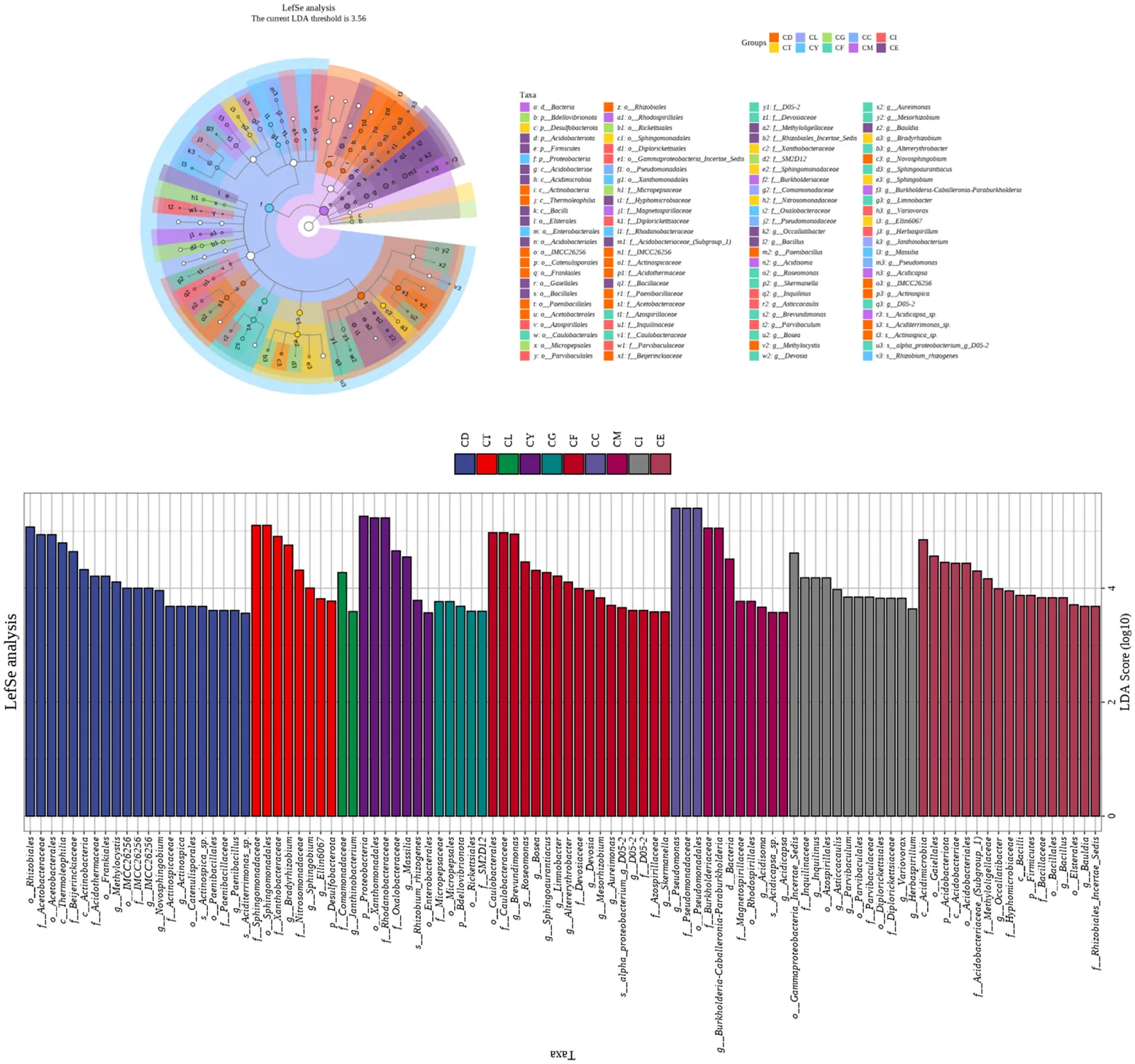
Biomarker bacteria identified by LEfSe across the 10 species, shown from phylum to genus level (LDA bar chart and cladogram; LDA > 2, *P* < 0.05).

Random forest classification corroborated the hierarchy of host effects. With species as the grouping factor, all 60 samples were assigned to their correct host species (overall accuracy = 100%; chance-level baseline = 10%). The 20 most important ASVs (Supplementary Figure S1) comprised 16 Proteobacteria, three Actinobacteriota, and one Acidobacteriota, and were dominated by the same genera that characterized the communities overall: *Roseiarcus* (the top-ranked ASV_144021 and ASV_12168), *Pseudomonas* (ASV_107166 and ASV_28504), *Sphingomonas* (ASV_35158), *Brevundimonas* (ASV_141379), *Reyranella* (ASV_133106), the *Burkholderia-Caballeronia-Paraburkholderia* complex (ASV_89614), *Conexibacter* (ASV_74201), and *Granulicella* (ASV_131253). Sixteen of these 20 lineages were also detected as biomarkers by LEfSe, whereas the two top-ranked *Roseiarcus* ASVs, *Reyranella* ASV_133106, and *Conexibacter* ASV_74201 were not, indicating that the two approaches are largely concordant but partly complementary.

Life form was classified with an out-of-bag accuracy of 86.7% and a cross-validation accuracy of 89.0% ± 9.1%; however, because each species represents a single life form, this performance largely reflects species recognition rather than a transferable life-form signature. Under leave-one-species-out cross-validation, life-form accuracy dropped to 50%, below the 60% majority-class baseline, with all replicates of the four terrestrial species misclassified as epiphytic (Supplementary Table S1). This indicates that the apparent life-form signal is largely carried by (and inseparable from) species-level differences in community composition.

### 3.6 Functional prediction of the communities

Functional prediction based on the MetaCyc database showed that, at Level 1, biosynthesis dominated the predicted functional profiles (69.8%), followed by degradation, utilization, and assimilation (15.1%) and the generation of precursor metabolites and energy (13.2%). At Level 2, the most abundant pathways were amino acid biosynthesis (17.2%); cofactor, prosthetic group, electron carrier, and vitamin biosynthesis (15.1%); nucleoside and nucleotide biosynthesis (13.3%); fatty acid and lipid biosynthesis (10.9%); and carbohydrate biosynthesis (5.7%; Figure 6). Between life forms, only three of the 20 major pathways differed significantly in relative abundance (adjusted *P* < 0.05): the non-oxidative branch of the pentose phosphate pathway was more abundant in epiphytic species (0.71% vs. 0.65%), whereas CDP-diacylglycerol biosynthesis I and II were both higher in terrestrial species (0.75% vs. 0.68%). Taxonomic composition varied sharply across species, yet the predicted core functional pathways barely changed between life forms: only three of the 20 major pathways differed. This points to pronounced functional redundancy within these communities.

**Figure 6:**
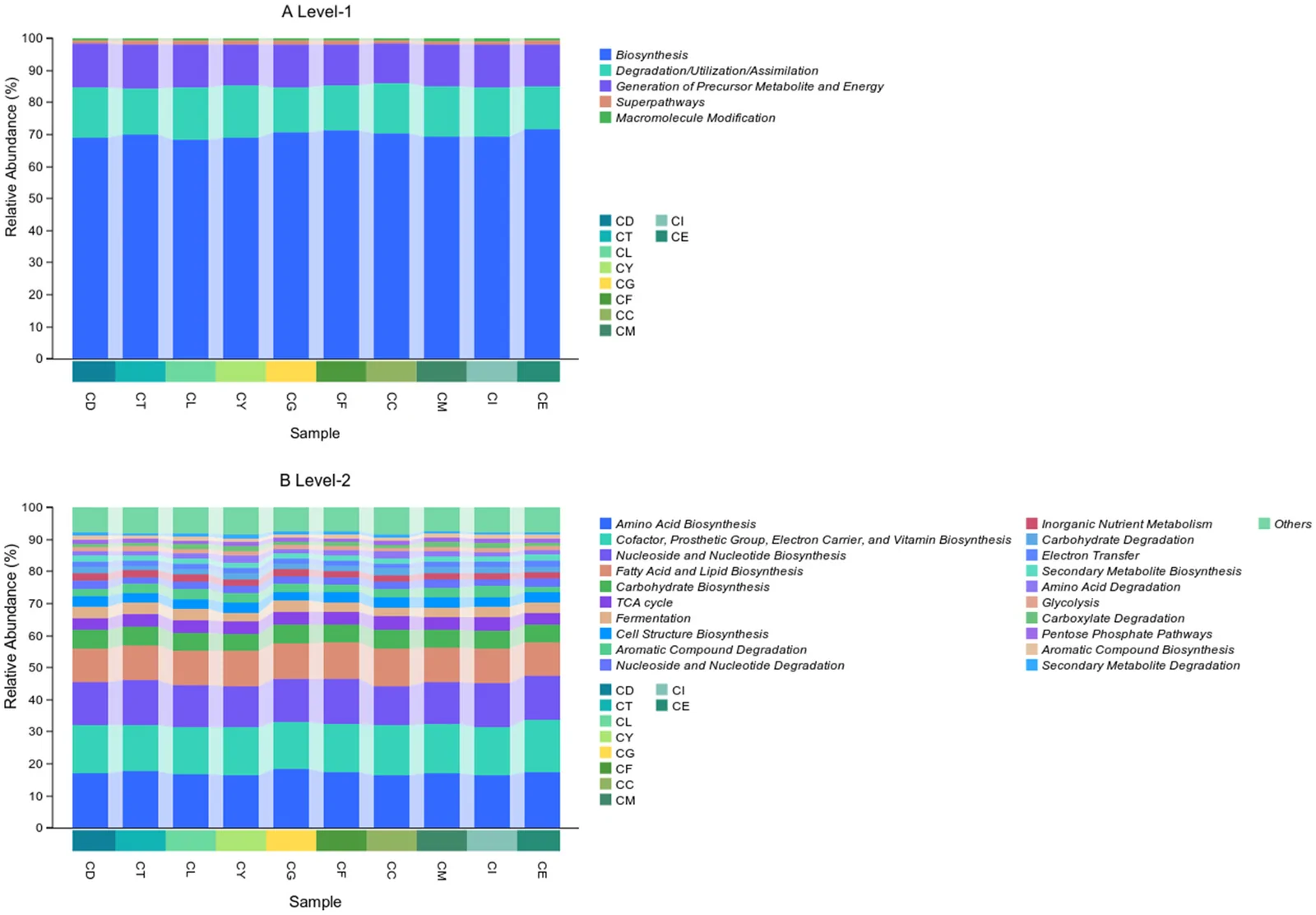
Predicted functional composition of root endophytic bacterial communities, inferred with PICRUSt2 against the MetaCyc database. **(A)** Relative abundance (%) of MetaCyc Level-1 categories. **(B)** Relative abundance (%) of the 20 most abundant Level-2 pathways, averaged within each host species. Values are expressed as a percentage of all predicted pathway copies in a sample, so categories sum to 100% within each panel. (Species codes CC-CY follow Table 1).

FAPROTAX assigned the ASVs to 55 ecological functional groups (Supplementary Table S3). Assignment success was uneven across samples. FAPROTAX requires genus-level resolution, which was reached for 70.7% of reads in terrestrial species but only 52.8% in epiphytic species. Summed guild counts reached 117.1% of non-singleton reads in terrestrial species and 69.5% in epiphytic species. The values exceed 100% because single ASVs were assigned to more than one guild. All abundances below are therefore expressed relative to the assigned pool.

Within the assigned pool, aerobic chemoheterotrophy was the most common guild in every sample, and its abundance was similar in the two life forms (37.57% in epiphytic vs. 39.87% in terrestrial species; BH-adjusted *P* = 0.50). Nitrogen fixation and nitrification also showed no life-form difference (8.85% vs. 5.74%, *P* = 0.061; and 1.73% vs. 1.10%, *P* = 0.83, respectively). Only four guilds differed between life forms. Three were more abundant in epiphytic species: cellulolysis (2.04% vs. 0.14%; *P* = 2.8 × 10^−5^), hydrocarbon degradation (0.98% vs. 0.12%; *P* = 0.014), and methylotrophy (1.18% vs. 0.29%; *P* = 0.032); one, denitrification, was more abundant in terrestrial species (0.10% vs. 0.03%; *P* = 0.0078) (Figure 7B). In contrast, all 19 major guilds differed significantly among the 10 species under both normalizations (Kruskal–Wallis, BH-adjusted *P* < 0.05; Figure 7A), consistent with the pattern of the MetaCyc pathways.

**Figure 7:**
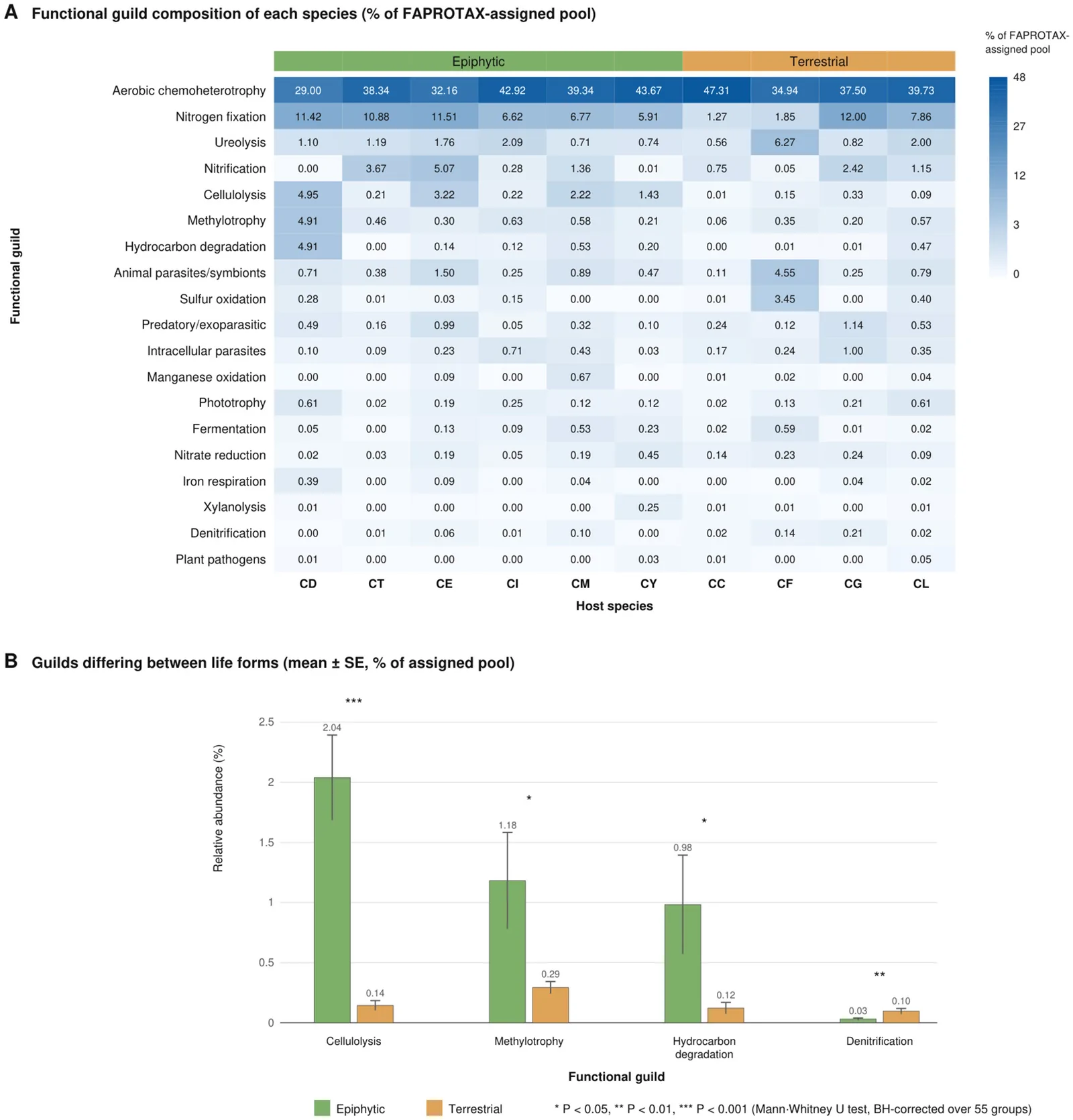
FAPROTAX-inferred ecological functional guilds of root endophytic bacteria. **(A)** Mean relative abundance (% of the FAPROTAX-assigned pool) of the 19 major guilds in each species (CC-CY follow Table 1). **(B)** Guilds differing significantly between life forms (mean ± SE; two-sided Mann–Whitney *U*-tests, Benjamini–Hochberg corrected across all 55 annotated groups; *P < 0.05, **P < 0.01, and ***P < 0.001). Denitrification refers to the same set of ASVs as nitrate denitrification, nitrite denitrification and nitrite respiration. Guilds are not mutually exclusive and do not sum to 100%.

### 3.7 Niche breadth and community assembly

The abundance-weighted niche breadth of dominant taxa was slightly higher in epiphytic (*B* = 5.89) than in terrestrial species (*B* = 5.54). Among all ASVs, 9,972 were classified as specialists with significant niche preference and 12,733 as generalists. The neutral community model explained only a small fraction of the relationship between ASV occurrence frequency and relative abundance (*R*^2^ = 0.035; community-level migration rate Nm = 220; Figure 8): of the 129,922 ASVs, 98.6% fell within the neutral prediction interval, 1.1% occurred above it and 0.2% below it. The normalized stochasticity ratio averaged 0.576 across the dataset and was nearly identical between epiphytic (0.579) and terrestrial (0.572) species, ranging from 0.38 to 0.77 among species.

**Figure 8:**
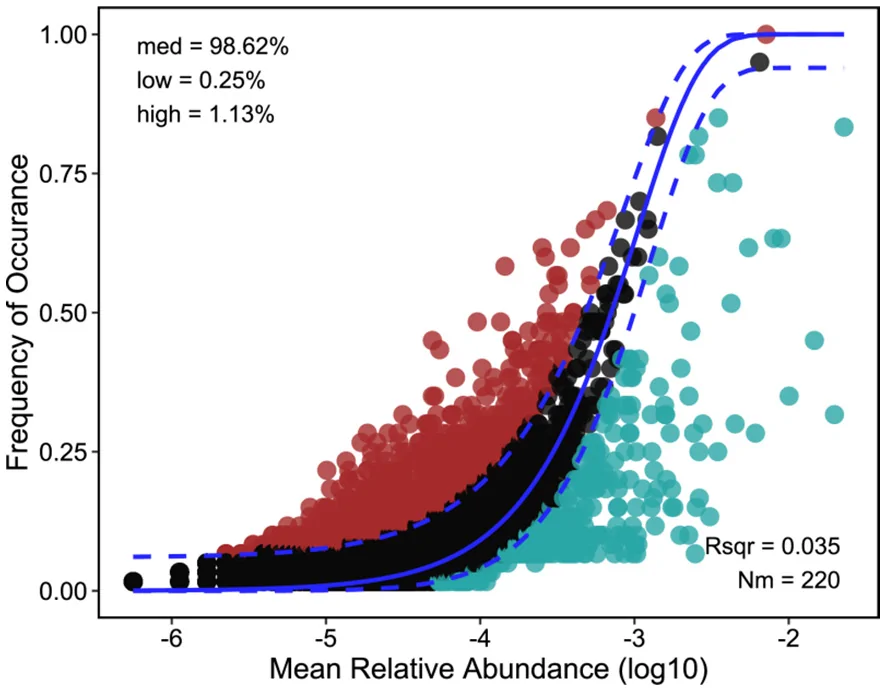
Fit of the Sloan neutral community model (*R*^2^ = 0.035, Nm = 220, and ASV level).

## 4 Discussion

Orchid microbiome research has long centered on mycorrhizal fungi, and how the root endophytic bacterial communities of *Cymbidium* vary with host life form and host species under controlled conditions has remained unknown. This study therefore aimed to (i) compare community composition and diversity among life forms, infrageneric sections and host species, (ii) identify the bacterial biomarkers of each species, (iii) predict the functional potential of these communities, and (iv) quantify the relative roles of deterministic selection and stochastic processes in community assembly. Grown under identical greenhouse conditions, the 10 species shared almost no core taxa; community structure differed most strongly at the species level (marginal PERMANOVA *R*^2^ = 0.599) and only weakly between life forms (*R*^2^ = 0.069); random forest classification assigned every sample to its host species; and assembly combined deterministic host selection among species with substantial stochasticity within species. Below, we discuss these findings against the available orchid literature, consider their practical implications for horticulture, the environment and agriculture, and outline the limitations of our approach and the future directions it opens.

### 4.1 Community composition and a minimal interspecific core microbiome

We profiled the root endophytic bacterial communities of 10 *Cymbidium* species grown in a single greenhouse. Proteobacteria dominated (mean 83.4%), followed by Actinobacteriota and Acidobacteriota. This pattern matches the tuber endophytic bacteria of *Gastrodia elata* (Zheng et al., 2023) and the root bacteria of cultivated *Paphiopedilum* (Cao et al., 2022), and is consistent with the broader dominance of Proteobacteria documented in the well-characterized *Arabidopsis* root microbiome (Bulgarelli et al., 2012). The leading genera were *Pseudomonas, Sphingomonas*, the *Burkholderia-Caballeronia-Paraburkholderia* complex, and *Bradyrhizobium*. Most of these taxa have documented growth-promoting and stress-resistance potential (Afzal et al., 2019; Saikia and Thakur, 2024). Pseudomonadaceae, Rhizobiaceae and Burkholderiaceae also rank among the dominant root endophytes of Cypripedium (Shan et al., 2024), and Pseudomonas strains are frequently recovered in culture-dependent orchid studies (Tsavkelova et al., 2006; Pavlova et al., 2017). High-throughput reports on the root endophytic bacteria of epiphytic orchids remain scarce, particularly from natural habitats. In the epiphyte *Dendrobium catenatum*, Proteobacteria likewise dominated across five cultivation regions (71.8–96.1% relative abundance), with *Pseudomonas, Sphingomonas*, and *Burkholderia* among the leading genera (Li et al., 2017), closely mirroring the profiles recovered here. Data from strictly natural-habitat epiphytes are still lacking, however, so how far proteobacterial dominance extends to wild epiphytic roots remains an open question.

The dataset contained 129,922 ASVs spread across 33 phyla and 779 genera, yet all 10 species shared only seven ASVs. Each of these seven appeared in at least one replicate of every species; presence in all 60 samples was not required. Only one ASV occurred in all 60 samples. A core microbiome is usually defined as the set of taxa that occur stably within a given host group (Lundberg et al., 2012). On that definition, the interspecific core of *Cymbidium* root endophytic bacteria is minimal, and host-specific members make up most of each community. This result holds only at exact-sequence resolution. It does not rule out a core at higher taxonomic ranks or in functional terms. *Pseudomonas* and *Sphingomonas*, for example, occurred at high frequency in most of the 10 species and may form a functional core shared across *Cymbidium*.

### 4.2 The effect of life form is significant but limited

Infrageneric grouping and life form were partially confounded in our design: all three species of section Jensoa are terrestrial, and all three of section Cyperochis are epiphytic, a limitation to keep in mind below. Between life forms, alpha-diversity indices were nearly identical, phylum-level abundances differed only as trends, PERMANOVA attributed just 6.9% of variation to life form, and niche breadth of dominant taxa was similar (5.89 vs. 5.54). Under identical cultivation, then, life form affects the *Cymbidium* root endophytic community, but only slightly.

Because of this confounding, part of the 6.9% may actually derive from differences among infrageneric groups. *Cymbidium* mycorrhizal work has shown that life-form shifts bring pronounced turnover of symbiotic fungi (Ogura-Tsujita et al., 2012); the bacterial effect here is plainly weaker. Telling whether bacteria and fungi follow different assembly rules, or whether cultivation simply homogenizes bacteria more, will take a balanced design sampling across life forms within one infrageneric group, plus validation on natural-habitat samples.

Root habitat should have mattered more than this. Epiphytic roots endure air exposure and periodic desiccation, whereas terrestrial roots sit in a stable substrate, so the two should in principle select different colonists. The contrast was muted, and the plant material explains why: every plant was a transplant raised in one greenhouse on the same substrate, so a shared source pool and uniform husbandry compressed microenvironmental differences. Cultivated orchids show the same pattern, with community structure tracking host factors such as growth stage more closely than environmental variation (Cao et al., 2022). One contrast did stand out: Actinobacteriota was richer in epiphytes (12.7% vs. 6.8%). Actinobacteria tolerate drought by forming spores and carry extensive secondary-metabolic and antagonistic machinery, so whether this reflects adaptation to exposed roots deserves testing on natural-habitat samples (Yadav et al., 2022). Because plants of both life forms were rooted in the same pot substrate under the same roof, they recruited from an essentially identical source pool. Cultivation experiments on *Dendrobium catenatum* support this interpretation: when the same species was grown attached to living trees or on cliffs rather than in pots, its root bacterial communities shifted significantly (Zhu et al., 2022). The uniform pot environment shared by our 10 species would therefore be expected to compress life-form contrasts, exactly as observed.

Random forest corroborated this: host species were recovered with perfect accuracy, whereas the life-form signal collapsed when species identity was held out (Section 3.5). The life-form effect therefore sits within species-specific differences and shows little sign of generalizing across species. Far from undermining the aim of this study, the weakness of the life-form effect is one of its central results: with environmental heterogeneity minimized, host species identity, not life form, emerges as the dominant correlate of community differentiation, which is precisely the question addressed by the title of this manuscript.

### 4.3 Host species is the dominant factor of community differentiation

Host species stood out at every level of analysis, even within this partially confounded design. In the marginal PERMANOVA models it explained 59.9% of community variation, against 35.6% for infrageneric group and 6.9% for life form, and species stayed sharply differentiated within a single group (*R*^2^ ≈ 0.51 in both Jensoa and Cyperochis). The species estimate also absorbs variation structured by life form and section, so it is not fully independent. The within-group contrasts escape that confounding and place most differentiation at the species level, beyond what infrageneric relatedness explains. Alpha diversity pointed the same way. Chao1 stayed flat across species, whereas Shannon, Simpson and Pielou evenness diverged highly significantly, so the species effect runs through evenness and dominance rather than richness: host filtering seems to promote or suppress the growth of colonists rather than cap how many taxa can colonize the roots. With Chao1 non-significant and six replicates per species, we treat the ranking of species as descriptive and do not formally compare high- with low-diversity species. More than half the tested genus-level taxa differed among species (321/590), and the 279 LEfSe biomarkers spanned all 10 species, each contributing enriched taxa of its own.

Host species identity is a major source of plant microbiome variation, well-documented across angiosperms (Fitzpatrick et al., 2018) and in model plants (Bulgarelli et al., 2012; Trivedi et al., 2020). All plants here came from one greenhouse under identical cultivation and a shared source pool, so environmental interference was largely removed. Strong differentiation still emerged, pointing to the plants themselves: interspecific differences in root exudate composition, root-surface morphology, colonization barriers, and immune recognition (Trivedi et al., 2020). Strong host selection also explains the minimal interspecific core, since few taxa colonize stably across species boundaries. Microbiome management must therefore be species-specific in *ex situ* conservation and artificial propagation. One inoculant formulation will not serve all species.

### 4.4 Relative roles of deterministic selection and stochastic processes in community assembly

The neutral community model (NCM) and the normalized stochasticity ratio (NST) yield statistical inferences about assembly processes, not direct demonstrations of ecological mechanisms. The NCM accounted for almost none of the frequency-abundance relationship (*R*^2^ = 0.035), so migration and drift alone cannot explain how Cymbidium root endophytic bacteria are distributed (Sloan et al., 2006). The overall NST mean of 0.576 sits just above the 0.5 threshold (Ning et al., 2019), leaving drift and dispersal limitation a considerable share of turnover in pairwise comparisons (Stegen et al., 2013). The two methods ask different questions. The NCM tests how far the overall frequency distribution departs from neutral expectation; NST weighs deterministic convergence against stochastic divergence at local scales (Zhou and Ning, 2017). The results need not conflict. Deterministic processes such as host selection drive community differentiation among species, whereas turnover among samples within a species is largely stochastic. NST also varied widely across species (0.38–0.77), so the strength of deterministic filtering depends on the host (Supplementary Figure S2). Niche breadth was broadly similar between the two life forms, and specialists and generalists coexisted in both (9,972 vs. 12,733 taxa, respectively), as the alpha-diversity and PERMANOVA results would predict.

### 4.5 Functional potential, application prospects, and limitations

Biosynthesis dominated the predicted metabolic profile (69.8%), with amino acid, cofactor and vitamin, and nucleoside and nucleotide biosynthesis the most enriched categories, as expected of endophytes that promote host growth and regulate host metabolism (Afzal et al., 2019; Saikia and Thakur, 2024). Culture-based work agrees: Cymbidium has yielded phosphate-solubilizing (Sun et al., 2025) and growth-promoting endophytes (Gontijo et al., 2018), and auxin synthesis by orchid-associated bacteria has been reported repeatedly (Tsavkelova et al., 2006, 2007; Pavlova et al., 2017). Community composition differed considerably among life forms and species, whereas predicted function barely did; only three major pathways separated the life forms. Functional redundancy is therefore pronounced: *Cymbidium* roots appear to select for bacterial metabolic functions, not for strict taxonomic identity.

FAPROTAX offers an independent check, because it assigns functions from taxonomy rather than from gene content and so shares few assumptions with the pathway predictions above. After correcting for the unequal assignable fractions, the dominant aerobic chemoheterotrophs and the nitrogen-cycling guilds did not differ between life forms, while every major guild differed among species. The exceptions track substrate, not host taxonomy: epiphytic roots carried more cellulolytic and hydrocarbon-degrading bacteria, terrestrial roots more denitrifiers. Canopy roots sit against bark-derived lignocellulose in an aerobic, oligotrophic matrix, whereas substrate roots encounter anoxic microsites more often, favoring anaerobic respiration.

The dominant genera (*Pseudomonas*, the *Burkholderia-Caballeronia-Paraburkholderia* complex, *Sphingomonas*, and *Bradyrhizobium*) and the species-specific biomarkers, such as *Pseudomonas* in Cymbidium cyperifolium and Burkholderiaceae in *C. mastersii*, are where targeted isolation of beneficial endophytes and inoculant development should start. The one ASV found in all 60 samples is the most direct lead to a core symbiont and should be isolated and identified first. All of them rest on 16S rRNA gene data alone, so any growth-promoting or symbiotic role still needs confirmation by strain isolation, cultivation and functional assays before practical use.

Several limitations apply. Functional profiles are 16S-based predictions (Langille et al., 2013), so key pathways and functional genes await metagenomic sequencing and strain isolation. The plants were greenhouse transplants, so whether the conclusions hold in natural habitats is untested; differing field conditions and source pools would probably amplify the life-form effect. Life form, infrageneric grouping and host species were partly confounded (Section 4.2), and the PERMANOVA *R*^2^ values are marginal effects, so balanced infrageneric sampling across life forms will be needed for fully independent estimates. Community typing also left a small cluster of terrestrial samples only: five of the six *Cymbidium cyperifolium* replicates and one of *C. faberi* (Section 3.4, Supplementary Figure S3). One species can evidently hold more than one community state, and seedling provenance and colonization history are worth tracking. Bacteria were the only kingdom profiled, so a complete picture of the *Cymbidium* root microbiome will need fungal data too, together with cross-kingdom interactions. A final limitation concerns the plants themselves, which came from natural populations rather than axenic tissue culture: colonists acquired before transplantation cannot be fully excluded. More than 5 years in a common substrate makes wholesale carry-over of habitat communities unlikely, however, and any residue would blur the species-level differences we report rather than create them. Common-garden trials from tissue-cultured plantlets would supply the fully controlled counterpart.

These limits aside, the results have practical implications. In horticulture, strong species specificity means inoculants for *Cymbidium* must be developed and tested species by species. The dominant genera (*Pseudomonas, Sphingomonas*, the *Burkholderia-Caballeronia-Paraburkholderia* complex, and *Bradyrhizobium*) and the species-specific biomarkers are priority targets for biofertilizer and biocontrol work; defined inocula may also improve survival and growth of micropropagated plantlets during acclimatization. Host-matched endophytes could improve establishment in *ex situ* conservation and reintroduction of endangered orchids. Agriculture is a longer step. Matching microbiome management to host genotype is a principle that speaks to crop breeding and cultivar-specific probiotics, but the taxa here are orchid-associated and would need dedicated testing in crops.

## 5 Conclusion

Ten *Cymbidium* species (six epiphytic, four terrestrial, and six replicates each) were grown in one greenhouse under identical cultivation, and their root endophytic bacterial communities were profiled by 16S rRNA gene amplicon sequencing. The conclusions below therefore apply to greenhouse-transplanted plants. Proteobacteria (83.4%), Actinobacteriota (10.3%), and Acidobacteriota (2.5%) dominated the communities, with *Pseudomonas, Sphingomonas*, the *Burkholderia-Caballeronia-Paraburkholderia* complex, *Bradyrhizobium*, and *Reyranella* as the main genera. The interspecific core microbiome was vanishingly small: seven ASVs shared across the 10 species, and one shared across all 60 samples. In the marginal single-factor models, host effects ranked as follows: life form (6.9%) < infrageneric group (35.6%) < host species (59.9%). The three factors were partly confounded in our design, so the *R*^2^ values are not independent. Species also stayed distinct within the same infrageneric group, which places most differentiation at the species level. The life-form effect was real but small, and this design cannot separate it from species and section effects. Predicted functional profiles were dominated by biosynthesis pathways and remained largely stable between life forms. At the inter-species scale, deterministic host selection drove community assembly, whereas stochastic processes contributed most to intra-species turnover. These findings provide a basis for mining beneficial endophytic bacteria, designing microbial inoculants, and supporting *ex situ* conservation and artificial propagation of *Cymbidium*. Future work should prioritize isolating and functionally verifying the candidate core taxa, extend the analysis to bacterial-fungal (multi-kingdom) interactions, and validate the present conclusions with natural-habitat samples.

## Data Availability

The raw 16S rRNA amplicon sequencing data generated in this study have been deposited in the NCBI Sequence Read Archive (SRA) under BioProject PRJNA1548608 and are accessible via the study accession SRP748239. The data comprise 60 paired-end Illumina NovaSeq 6000 runs (accession numbers SRR41314197 to SRR41314256). Associated BioSample records (SAMN64353438–SAMN64353497) provide sample-level metadata.
