# Supplementary material for: Host species identity outweighs life form in shaping the root endophytic bacterial communities of 10 Cymbidium species grown under uniform conditions
Source: Front Microbiol. 2026 Oct 29;17:1996961. doi: 10.3389/fmicb.2026.1996961 (PMC13758987; doi:10.3389/fmicb.2026.1996961)
Supplement: Supplementary file 1 [file Supplementary_file_1.docx]

**Supplementary Material**

Supplementary Figure S1. Random-forest classification of host species from root endophytic bacterial community composition. Bars show the 20 ASVs with the highest feature importance in the species classifier (500 trees; out-of-bag accuracy 100%), labeled with the ASV ID and its finest taxonomic assignment; the 20 ASVs comprised 16 Proteobacteria, three Actinobacteriota and one Acidobacteriota, and 16 of them were also detected as biomarkers by LEfSe (Section 3.5). Model settings are given in Section 2.4.

Supplementary Figure S2. Normalized stochasticity ratio (NST) of the root endophytic bacterial communities of the ten *Cymbidium* species (species codes follow Table 1). The distribution of pairwise NST values within each species (six replicates) is shown; values below and above the empirical threshold of 0.5 indicate predominantly deterministic and predominantly stochastic assembly, respectively (Ning et al., 2019). Species-level means ranged from 0.38 to 0.77, with an overall mean of 0.576.

Supplementary Figure S3. Bacterial community typing of the 60 root samples. Clustering on Bray-Curtis distances supported an optimum of two community types (Calinski-Harabasz index; 54 and 6 samples, respectively). Bars show the community composition of individual samples, grouped by cluster and host species (species codes follow Table 1). Cluster 2 comprised only terrestrial samples—five of the six *Cymbidium cyperifolium* replicates and one *C. faberi* replicate; all samples of the other eight species fell into Cluster 1 (Section 3.4).

Supplementary Table S1. Taxonomic annotation and occurrence of the seven core ASVs shared by all ten *Cymbidium* species. An ASV was considered shared if it was detected in at least one replicate sample of each of the ten species; occurrence in all 60 samples was not required.

| **ASV ID** | **Phylum** | **Class** | **Order** | **Family** | **Genus** | **Samples** **detected** **(n/60)** | **Total** **reads** | **Mean relative** **abundance (%)** |
| --- | --- | --- | --- | --- | --- | --- | --- | --- |
| ASV_86589 | Proteobacteria | Gammaproteobacteria | Pseudomonadales | Pseudomonadaceae | *Pseudomonas* | 50 | 40,578 | 2.28 |
| ASV_22104 | Proteobacteria | Alphaproteobacteria | Rhizobiales | Xanthobacteraceae | *Bradyrhizobium* ^a^ | 60 | 12,694 | 0.71 |
| ASV_35699 | Proteobacteria | Alphaproteobacteria | Rhizobiales | Xanthobacteraceae | *Bradyrhizobium* | 57 | 11,522 | 0.65 |
| ASV_16347 | Proteobacteria | Alphaproteobacteria | Reyranellales | Reyranellaceae | *Reyranella* | 51 | 6,219 | 0.35 |
| ASV_73514 | Proteobacteria | Gammaproteobacteria | Burkholderiales | Burkholderiaceae | *Burkholderia-Caballeronia-Paraburkholderia* | 49 | 4,644 | 0.26 |
| ASV_154881 | Proteobacteria | Gammaproteobacteria | Burkholderiales | Comamonadaceae | Unclassified ^b^ | 47 | 4,410 | 0.25 |
| ASV_115365 | Proteobacteria | Alphaproteobacteria | Rhizobiales | Beijerinckiaceae | *Roseiarcus* | 49 | 2,500 | 0.14 |

a ASV_22104 was the only ASV detected in all 60 samples.

b ASV_154881 could not be assigned below the family level (Comamonadaceae).

Supplementary Table S2. Random forest classification of life form: overall performance and per-species results under leave-one-species-out cross-validation (LOGO-CV).

A. Overall performance of life-form classification

| **Validation scheme** | **Accuracy** |
| --- | --- |
| Out-of-bag (OOB) estimate | 86.7% |
| Six-fold stratified cross-validation (five repeats) | 89.0% ± 9.1% |
| Leave-one-species-out cross-validation (LOGO-CV) | 50.0% (30/60) |
| Majority-class baseline a | 60.0% (36/60) |

B. Per-species results under LOGO-CV

| **Held-out species** | **Life form** | **Correctly classified replicates** | **Accuracy (%)** | **Misclassified replicates predicted as** |
| --- | --- | --- | --- | --- |
| CC | terrestrial | 0/6 | 0.0 | epiphytic (6/6) |
| CD | epiphytic | 6/6 | 100 | – |
| CE | epiphytic | 6/6 | 100 | – |
| CF | terrestrial | 0/6 | 0.0 | epiphytic (6/6) |
| CG | terrestrial | 0/6 | 0.0 | epiphytic (6/6) |
| CI | epiphytic | 6/6 | 100 | – |
| CL | terrestrial | 0/6 | 0.0 | epiphytic (6/6) |
| CM | epiphytic | 6/6 | 100 | – |
| CT | epiphytic | 0/6 | 0.0 | terrestrial (6/6) |
| CY | epiphytic | 6/6 | 100 | – |
| **Overall** |  | **30/60** | **50.0** |  |

Species codes (CC–CY) follow Table 1 of the main text.

Supplementary Table S3. FAPROTAX-inferred ecological functional groups of root endophytic bacteria: relative abundance under two normalisations, and tests for differences between life forms. Norm A expresses relative abundance as a percentage of all non-singleton reads; Norm B expresses relative abundance as a percentage of the FAPROTAX-assigned pool and is the normalisation used in Figure 7 and in the main text. *P* values are from two-sided Mann-Whitney U tests between life forms, Benjamini-Hochberg corrected across all 55 annotated groups. The last column marks the guilds displayed in Figure 7A. Guilds are not mutually exclusive and need not sum to 100%.

| **Rank** | **Functional group** | **Norm A: mean %** | **Norm A: epiphytic %** | **Norm A: terrestrial %** | **Norm B: mean %** | **Norm B: epiphytic %** | **Norm B: terrestrial %** | **P (BH, 55 tests)** | **Higher in** | **In Figure 7** |
| --- | --- | --- | --- | --- | --- | --- | --- | --- | --- | --- |
| 1 | chemoheterotrophy | 35.399 | 26.994 | 48.008 | 39.588 | 38.959 | 40.531 | 0.569 | terrestrial |  |
| 2 | aerobic_chemoheterotrophy | 34.475 | 25.942 | 47.274 | 38.491 | 37.572 | 39.87 | 0.503 | terrestrial | yes |
| 3 | nitrogen_fixation | 6.003 | 6.438 | 5.35 | 7.608 | 8.851 | 5.744 | 0.0613 | epiphytic | yes |
| 4 | ureolysis | 1.792 | 0.914 | 3.11 | 1.724 | 1.266 | 2.41 | 0.569 | terrestrial | yes |
| 5 | nitrification | 1.235 | 1.366 | 1.039 | 1.476 | 1.731 | 1.095 | 0.828 | epiphytic | yes |
| 6 | aerobic_ammonia_oxidation | 1.23 | 1.357 | 1.039 | 1.469 | 1.718 | 1.095 | 0.758 | epiphytic |  |
| 7 | animal_parasites_or_symbionts | 1.051 | 0.44 | 1.969 | 0.991 | 0.702 | 1.426 | 0.596 | terrestrial | yes |
| 8 | human_associated | 1.051 | 0.44 | 1.968 | 0.991 | 0.702 | 1.426 | 0.596 | terrestrial |  |
| 9 | human_pathogens_all | 1.051 | 0.44 | 1.967 | 0.991 | 0.702 | 1.425 | 0.596 | terrestrial |  |
| 10 | cellulolysis | 0.77 | 1.192 | 0.138 | 1.281 | 2.039 | 0.144 | 2.75e-05 | epiphytic | yes |
| 11 | methylotrophy | 0.669 | 0.9 | 0.323 | 0.826 | 1.182 | 0.292 | 0.0321 | epiphytic | yes |
| 12 | dark_oxidation_of_sulfur_compounds | 0.537 | 0.059 | 1.254 | 0.433 | 0.078 | 0.965 | 0.126 | terrestrial | yes |
| 13 | hydrocarbon_degradation | 0.476 | 0.718 | 0.115 | 0.639 | 0.983 | 0.122 | 0.0138 | epiphytic | yes |
| 14 | methanotrophy | 0.474 | 0.714 | 0.113 | 0.635 | 0.978 | 0.12 | 0.0138 | epiphytic |  |
| 15 | predatory_or_exoparasitic | 0.321 | 0.216 | 0.478 | 0.413 | 0.35 | 0.507 | 0.392 | terrestrial | yes |
| 16 | intracellular_parasites | 0.278 | 0.172 | 0.438 | 0.336 | 0.265 | 0.442 | 0.0762 | terrestrial | yes |
| 17 | methanol_oxidation | 0.195 | 0.186 | 0.21 | 0.191 | 0.204 | 0.172 | 0.435 | epiphytic |  |
| 18 | human_pathogens_pneumonia | 0.184 | 0.172 | 0.202 | 0.239 | 0.254 | 0.217 | 0.41 | epiphytic |  |
| 19 | phototrophy | 0.18 | 0.149 | 0.227 | 0.227 | 0.219 | 0.241 | 0.828 | terrestrial | yes |
| 20 | photoheterotrophy | 0.18 | 0.149 | 0.227 | 0.227 | 0.219 | 0.24 | 0.828 | terrestrial |  |
| 21 | fermentation | 0.146 | 0.09 | 0.23 | 0.167 | 0.172 | 0.16 | 0.0643 | epiphytic | yes |
| 22 | nitrate_reduction | 0.132 | 0.089 | 0.196 | 0.163 | 0.155 | 0.174 | 0.25 | terrestrial | yes |
| 23 | nitrate_respiration | 0.076 | 0.034 | 0.138 | 0.088 | 0.062 | 0.126 | 0.0762 | terrestrial |  |
| 24 | nitrogen_respiration | 0.076 | 0.034 | 0.138 | 0.088 | 0.062 | 0.126 | 0.0762 | terrestrial |  |
| 25 | dark_thiosulfate_oxidation | 0.055 | 0.003 | 0.134 | 0.053 | 0.003 | 0.128 | 0.106 | terrestrial |  |
| 26 | nitrite_respiration | 0.055 | 0.02 | 0.107 | 0.063 | 0.037 | 0.103 | 0.00784 | terrestrial |  |
| 27 | denitrification | 0.05 | 0.017 | 0.1 | 0.057 | 0.031 | 0.096 | 0.00784 | terrestrial |  |
| 28 | nitrate_denitrification | 0.05 | 0.017 | 0.1 | 0.057 | 0.031 | 0.096 | 0.00784 | terrestrial |  |
| 29 | nitrite_denitrification | 0.05 | 0.017 | 0.1 | 0.057 | 0.031 | 0.096 | 0.00784 | terrestrial |  |
| 30 | manganese_oxidation | 0.044 | 0.062 | 0.017 | 0.082 | 0.127 | 0.016 | 0.828 | epiphytic | yes |
| 31 | anoxygenic_photoautotrophy | 0.041 | 0.019 | 0.074 | 0.052 | 0.034 | 0.078 | 0.0665 | terrestrial |  |
| 32 | photoautotrophy | 0.041 | 0.019 | 0.074 | 0.052 | 0.034 | 0.078 | 0.0665 | terrestrial |  |
| 33 | anoxygenic_photoautotrophy_S_oxidizing | 0.04 | 0.018 | 0.074 | 0.05 | 0.031 | 0.078 | 0.0364 | terrestrial |  |
| 34 | iron_respiration | 0.036 | 0.051 | 0.013 | 0.06 | 0.09 | 0.015 | 0.276 | epiphytic | yes |
| 35 | aromatic_compound_degradation | 0.028 | 0.014 | 0.049 | 0.031 | 0.021 | 0.045 | 0.181 | terrestrial |  |
| 36 | xylanolysis | 0.018 | 0.025 | 0.008 | 0.028 | 0.043 | 0.007 | 0.69 | epiphytic | yes |
| 37 | dark_hydrogen_oxidation | 0.016 | 0.005 | 0.033 | 0.015 | 0.008 | 0.025 | 0.106 | terrestrial |  |
| 38 | chitinolysis | 0.012 | 0.001 | 0.027 | 0.011 | 0.001 | 0.027 | 0.0237 | terrestrial |  |
| 39 | plant_pathogen | 0.008 | 0.003 | 0.016 | 0.011 | 0.007 | 0.016 | 0.106 | terrestrial | yes |
| 40 | chlorate_reducers | 0.006 | 0.005 | 0.007 | 0.008 | 0.009 | 0.005 | 0.532 | epiphytic |  |
| 41 | aerobic_nitrite_oxidation | 0.005 | 0.009 | 0 | 0.007 | 0.012 | 0 | 0.0643 | epiphytic |  |
| 42 | nitrate_ammonification | 0.003 | 0.002 | 0.005 | 0.005 | 0.005 | 0.004 | 0.888 | epiphytic |  |
| 43 | nitrite_ammonification | 0.003 | 0.002 | 0.005 | 0.005 | 0.005 | 0.004 | 0.888 | epiphytic |  |
| 44 | fumarate_respiration | 0.002 | 0.002 | 0.002 | 0.004 | 0.005 | 0.003 | 0.596 | epiphytic |  |
| 45 | knallgas_bacteria | 0.002 | 0.002 | 0.002 | 0.004 | 0.005 | 0.003 | 0.596 | epiphytic |  |
| 46 | aromatic_hydrocarbon_degradation | 0.002 | 0.002 | 0.002 | 0.003 | 0.003 | 0.002 | 0.569 | epiphytic |  |
| 47 | dark_sulfide_oxidation | 0.001 | 0.002 | 0 | 0.001 | 0.002 | 0 | 0.503 | epiphytic |  |
| 48 | dark_sulfur_oxidation | 0.001 | 0.002 | 0 | 0.001 | 0.002 | 0 | 0.503 | epiphytic |  |
| 49 | ligninolysis | 0.001 | 0 | 0.003 | 0.001 | 0 | 0.002 | 0.392 | terrestrial |  |
| 50 | anoxygenic_photoautotrophy_H2_oxidizing | 0.001 | 0.002 | 0 | 0.002 | 0.003 | 0 | 0.191 | epiphytic |  |
| 51 | aliphatic_non_methane_hydrocarbon_degradation | 0.001 | 0 | 0.002 | 0.001 | 0 | 0.002 | 0.0897 | terrestrial |  |
| 52 | human_gut | 0 | 0 | 0.001 | 0 | 0 | 0.001 | 0.178 | terrestrial |  |
| 53 | mammal_gut | 0 | 0 | 0.001 | 0 | 0 | 0.001 | 0.178 | terrestrial |  |
| 54 | nonphotosynthetic_cyanobacteria | 0 | 0 | 0 | 0 | 0 | 0 | 0.828 | equal |  |
| 55 | dark_iron_oxidation | 0 | 0 | 0 | 0 | 0 | 0 | 0.569 | equal |  |
